# Multiplicity: An Explorative Interview Study on Personal Experiences of People with Multiple Selves

**DOI:** 10.3389/fpsyg.2017.00938

**Published:** 2017-06-13

**Authors:** Gergő Ribáry, László Lajtai, Zsolt Demetrovics, Aniko Maraz

**Affiliations:** Department of Clinical Psychology and Addiction, Institute of Psychology, Eötvös Loránd UniversityBudapest, Hungary

**Keywords:** identity, dissociative identity disorder, identity integration, self concept, personality disorders

## Abstract

**Background and aims:** Personality psychology research relies on the notion that humans have a single self that is the result of the individual's thoughts, feelings, and behaviors that can be reliably described (i.e., through traits). People who identify themselves as “multiple” have a system of multiple or alternative, selves, that share the same physical body. This is the first study to explore the phenomenon of multiplicity by assessing the experiences of people who identify themselves as “multiple.”

**Methods:** First, an Internet forum search was performed using the terms “multiplicity” and “multiple system.” Based on that search, people who identified themselves as multiple were contacted. Interviews were conducted by a consultant psychiatrist, which produced six case vignettes.

**Results:** Multiplicity is discussed on Twitter, Tumblr, Google+ and several other personal websites, blogs, and forums maintained by multiples. According to the study's estimates, there are 200–300 individuals who participate in these forums and believe they are multiple. Based on the six interviews, it appears that multiples have several selves who are relatively independent of each other and constitute the personality's system. Each “resident person” or self, has their own unique behavioral pattern, which is triggered by different situations. However, multiples are a heterogeneous group in terms of their system organization, memory functions, and control over switching between selves.

**Conclusions:** Multiplicity can be placed along a continuum between identity disturbance and dissociative identity disorder (DID), although most systems function relatively well in everyday life. Further research is needed to explore this phenomenon, especially in terms of the extent to which multiplicity can be regarded as a healthy way of coping.

## Introduction

People usually have an alternating set of behaviors triggered by various social roles and different social events. The same person can be a mother, a friend, an employee, a committed vegetarian, a frustrated public transport user and many other identities, often at the same time. Nevertheless, these roles and behaviors accumulate into one unified self. Thus, people experience a relatively undivided, continuous identity, in which roles are intertwined with one another. In contrast, those people who identify themselves as “multiple” believe that they do not have just one “true” self and that they possess a multiple number of different selves who are all important and take turns controlling the behavior. These “selves” each have their own thoughts, desires, interests, and histories. Although the idea that each person possesses, to some extent, several “subselves” is not novel, this is the first paper to explore people who call themselves “multiple” through Internet forums and interviews. Unlike previous studies on multiplicity, this paper proposes that “multiplicity” is an extreme form of identity splitting, which often encompasses individuals with features of dissociative identity disorder (DID).

Most personality psychology research relies on the notion that humans have a single self that is the result of the individual's thoughts, feelings, and behaviors that can be described (i.e., through traits). This idea has been challenged by many theorists over the past 60 years. Jung (1953) was the first to describe systematically the origin of the selves, which can arise from personal experiences (e.g., as a result of trauma) or can develop from stereotyped roles, such as the role of a teacher or a famous person. Selves can become the “persona” or the “shadow” or can be present in the form of an “archetype,” which altogether construct the personality. Angyal (1973) postulated that the mind is made up of subsystems that interact with one another and can result in mental pressure, intrusion or invasion in the case of conflicting interests. Mitchell (1993), following the object-relation theory approach, proposed that objects that were introjected early in life become “self statuses” and lead a life of their own within the personality. More recently, Lester (2012) proposed the term “subselves” or “multiple selves” to argue that the concept of a single self is oversimplistic. This idea followed the theory by Carter (2008), who distinguished selves into major, minor, and fragmentary “micro-selves” within one person.

Multiplicity refers to people who behave as if they have at least two distinct selves, which are believed to be socially constructed (Spanos, 1994). Each self has his or her own thoughts, emotional reactions, preferences, behavior, and even memory. Often, the only shared entity is the physical body they live in. Thus, in the current study, multiplicity is a term that encompasses extreme splitting of the personality, which is qualitatively different from most people's everyday experience.

Empirical measurement of multiplicity is sparse. The first inventory published in the field was the Plural Self Scale (Altrocchi, 1999), which assesses the structure of the personality. High scores indicate that thoughts and feelings are different through time and situations. The other inventory to assess multiplicity was developed by Carter (2008) and consists of 20 items, such as “Does your handwriting change noticeably at different times?” or “Do you swing suddenly from one mood to another for no apparent reason?.” However, these scales measure the integrity of the self and rely on the assumption that there is a “you” or “I” who is able to self-reflect. Individuals who consider themselves multiple refer to themselves as a group of selves (“we”). Thus, questionnaires that assess the extent of self-integration fail to assess the experience of individuals who claim to have multiple selves who all have different thoughts, feelings, motivations, and levels of complexity.

In addition to issues of nosology, it is unclear how a stable self relates to self-esteem and well-being. Some authors argue that the more stable the self is, the better the psychological functioning and adjustment (Donahue et al., 1993; Kernis, 2005). In contrast, other authors argue that an undifferentiated self may be too rigid to adapt to the different expectations of the complex requirements of a social life; thus, the key to a successful social life is the high level of differentiation of self-concepts (Gergen, 1971; Linville, 1987). Therefore, multiplicity may be the key factor in exploring the relation between the stability of self and well-being.

Multiplicity shares common features with identity disturbance and its more severe form, DID. DID is considered to be a set of disrupted functions of consciousness, memory, identity, or perception of the environment that often serves as a way of coping with extreme stress and often originates from trauma (Dalenberg et al., 2012; Lynn et al., 2012). Trauma vs. non-trauma related (neutral) personality states are associated with different areas of cerebral blood flow (determined using positron emission tomography) in female patients with DID, which supports the notion that DID patients have different autobiographical selves (Reinders et al., 2003). However, the concept of DID is far from being a consensual diagnostic category, and some authors suggest that DID can be explained by a tendency toward high fantasy and motivated role-playing (Piper and Merskey, 2004; Reinders et al., 2012).

Individuals with multiplicity also have unique features that do not seem to fit existing mental health categories. The Multiplicity Scale (Carter, 2008) incorporates many items that refer to dissociation, and one study using this instrument found that the level of personality integrity was associated only with neuroticism (*r* = 0.35, marginally significant) among the Big Five factors in a sample of undergraduate students (*N* = 67; Lester and Carter, 2013), which is in line with other studies that have found an association between dissociation and neuroticism (*r* = 0.27; Ruiz et al., 1999). Given that dissociation, especially in the form of multiplicity, is rare, a sample of undergraduates may not reveal meaningful information about people who believe they have multiple selves. No studies were found that assessed multiplicity as an extreme form of splitting. Since self-concept has broader implications in social adjustment and mental health, understanding how the structure of personality relates to functioning beyond measures of association merits closer examination.

Given the lack of existing research, we aimed to explore multiplicity via Internet forums and, subsequently, interviews with six multiples. Our results aim to help determine whether multiplicity is an extreme (maladaptive) form of identity disturbance. Furthermore, our results may enable a deeper understanding of the concepts of self and identity in relation to mental health.

## Materials and methods

We attempted to assess multiplicity via an Internet forum search, questionnaires, and interviews. However, individuals tended to refuse to answer the survey questions, claiming that every self within the system had a different age, gender, and behavior, and they could not simply report the “average,” as this would be biased reporting. Thus, we decided to omit the responses of the self-reported questionnaires and include only the findings of the forum search and interviews.

### Internet forums

In order to explore multiplicity in the online environment, a free-text search was applied using the terms “multiplicity” and “multiple system.” Given that he search listing was based largely on relevance and the number of clicks, we expected to find the most active Internet forums on multiplicity using this method. Additional blogs and forums were discovered through the located forums because multiples tend to cross-reference each other's blog posts. The forum search aimed to contextualize the phenomenon and understand the unique terminology that multiples use to name their experiences and communicate with each other.

### Interviews

Based on the forum search, people who identified themselves as multiples on the forums were contacted directly or via a call placed on our Tumblr and Google+ pages. As a result, nine people agreed to be interviewed via online video or voice call. Three people did not respond to our subsequent letters or canceled the interviews at the last minute. In the end, interviews were conducted with six people. One multiple agreed to a second interview with another “alter” (resident person). Participants were told that scientific research was being conducted on multiplicity and that they would not be rewarded for participation. Interviews were 23–70 min long. Their real names and pseudo-names were changed, and personal information was removed from this article to protect their identities.

Interviews were conducted by a consultant psychiatrist. Conversations were not recorded for ethical reasons, and the study relied on notes taken by two assistants during the interviews. Interview questions were selected with the aim of exploring the person's background and psychiatric condition, especially regarding the symptoms of dissociation. Thus, questions were selected based on psychiatric first interviews (demographic information, previous treatment, and diagnoses), our Internet forum search on multiplicity (number of resident persons, cooperation between resident persons, reasons for switching, and illness insight) and DID symptoms. See Appendix 1 in Supplementry Material for the interview questions. The interview was expected to last for about 30 min in order not to overburden the participants and trigger potentially severe dissociative symptoms.

The study protocol was approved by the Institutional Review Board of Eötvös Loránd University.

## Results

### Internet forums

Based on our Internet search, multiplicity is present on Twitter, Tumblr, Google+ and several other personal websites, blogs, and forums maintained by multiples. We read about 30 multiples' blogs, in which they often referred to several other online and offline multiple systems. Based on our estimate, there are 200–300 individuals who participate in these forums and believe they are multiple, although it is difficult to ascertain the valid prevalence.

A multiple may maintain a blog or an account for the entire system or for only one resident person within the system. Multiples use the Internet to connect with each other and to share their experiences. Recurring themes include sharing their daily experiences and their philosophical and psychological efforts to describe their personal experiences, fear of discrimination and experiences of harassment. They challenge cultural norms and question the labeling of multiplicity as a mental disorder. Typically, multiples use their unique terminology to refer to common experiences, for example, “system” (which is the term used to refer to themselves, i.e., a system of persons), “resident persons” (or “alters,” who are alternative personalities sharing the body), “fronting” (when one resident person takes control over the behavior in a particular moment or period of time) and “host” (the original personality, often the one who has been present from birth).

Sometimes they describe themselves and their residents as rooted in a fantasy world, like the heroes in animation movies. It is possible, for example, for a resident person to change in age, whereas other residents can be “otherkin” (a conscious thing that is not human), which can vary between a mythological fairy to an entire planet. Most systems do not report amnesic barriers or recall traumatic events, and they insist that their multiplicity is something they were born with. Many of them call this healthy or a natural state of identity.

Multiples are a heterogeneous group. According to their posts, their triggers, how they switch between resident persons and the way they organize real-life behaviors differ greatly from one system to another.

A recurring topic on forums is the gender identity of multiples. Many multiples experience transgender issues and gender dysphoria because their residents have different genders and sexual preferences. There are many similarities between multiplicity and lesbian, gay, bisexual, transgender, and queer (LGBTQ) rights activism. Multiplicity uses many of the terms that the LGBTQ community uses, such as “coming out,” which means “revealing themselves” to the outside world as a multiple.

### Case vignettes

For reasons of clarity, participants are referred to as he or she, even though they identify themselves as “we.” We relied on our best judgment to determine their sex based on voice or picture, which was often different from the gender with which they identified. Interviews were shortened for publication.

#### Vignette 1—Dylan

A 19-year-old system presented herself as Kerry. The body had a female voice and look in the video call. She lives in a small town with her family and studies psychology at a university.

She reported being the “host of a multiple system,” which consists of eight alters among whom she is the main person. The alters know about each other and have good relations, except one who thinks the others do not exist and are only made up. Kerry stated that she does not usually remember events when she is not fronting.

The system was born when she was about 12–13 years old. She reported that the appearance of another resident person typically is triggered by stressful situations. For example, one resident person is afraid of dogs, which can trigger a switch to another resident who is less afraid of them. However, the switch can occur randomly without an obvious trigger. Fronting of a resident person can last from minutes to several days. She described one resident person called Wotany, who is a female, approximately the same age as she. Wotany has long hair and is very feminine, compared to Kerry. Wotany is very motherly, according to Kerry, and they get along well. One time, Wotany took over Kerry during a test, and as a result, the handwriting changed. This led to Kerry having to tell the professor about her condition.

She stated that she used to hear several voices and admittedly “lost time,” referring to a more unstable period in her life. As a result, she was diagnosed with DID. Currently, she is on psychiatric medication (escitalopram), which she finds helpful. At a later stage of the interview, she added that she still sometimes hears voices, which bothers her.

#### Vignette 2—space system

A 23-year-old Caucasian woman (female voice) introduced herself as Matthew. She lives in an East Coast college town with her fiancé. She currently is unemployed.

She described herself as the host of the “Space system” and explained that she is the central (dominant) person in the system. She stated, “We are a bunch of weirdos. Other people do not believe we exist.” There are approximately 20 members in the system. Most of the resident persons are fictional characters, like Luke Skywalker and Han Solo, from seven or eight various sources, such as TV shows, movies, and classical literature. Within the system, there are two subsystems. Loss of control is rare.

She described the system as a large community of housemates: “Being a system feels like having tenants …sharing mind and body space with a bunch of people with whom you may or may not get on well.” They have a “headspace” where everyone “hangs out.” There are frequent (verbal) interactions, even arguments among residents. She reported that the switches happen consciously, although they also may occur unexpectedly. To switch, she closes her eyes and moves her mind backwards and another person floats in. Switches also can be random, typically when someone new appears in the system and makes his or her own introduction.

She stated that her system has been around since she was 6 years old, following a traumatic event that she preferred not to share with us. However, she also stated that she was 12 when she noticed that there were other people living in her body at the same time. After the discovery, the first resident person, who was abusive, made her hurt herself, and it took her some time to come to terms with this person. By now, they have established house rules and have made peace with each other. The system, approximately as it is now, had developed by the age of 16. At that time, she was still in an abusive situation, but that ended at the age of 18.

She has seen specialists for her mental health issues. She was diagnosed with post-traumatic stress disorder (PTSD) and generalized anxiety disorder (GAD), but she believes that she also has bipolar disorder and borderline personality disorder. She also was diagnosed with dissociative disorders not otherwise specified, but she believes that she has DID. She does not think that multiplicity itself is an illness, but thinks that PTSD and disordered communication are issues that need treatment. Currently, she takes a mood stabilizer, which she finds helpful.

#### Vignette 3—Sarah and Jamie

Sarah is a 23-year-old woman who lives in a major city. She studied to become a writer, but paused her studies due to financial difficulties.

She described her system as consisting of two members: Sarah and her brother, Jamie. “We are both pretty normal people,” she stated. “We know all about each other. We can remember exactly what the other says or does. In fact, we are very organized people.” She reported that they have different values, opinions, tastes, and social life. They have different facial expressions and walk differently, and their voices sound different as well. They can both be present at the same time, but they avoid doing so because it confuses other people. They are only present together at the same time when they both find it enjoyable.

They switch several times a day: “We decide when to switch; we make schedules, who gets which hours. Rarely do we switch unintentionally. An unintended switch can only occur when one deals with stress.” She explained that both of them have been present since childhood, but neither of them knows when the other one first appeared. At the age of 12, Sarah was sexually assaulted. Following this incident, she left and only come back 6 years later. Jamie is now a female-to-male transsexual. Sarah says, “If he had been alone, he would have chosen surgery.” The decision about hormone treatment took two years to make, and he had no external help. He started to transform physically after 6 months of hormonal treatment, which bothered Sarah; therefore, he has stopped taking the medication for now.

Sarah stated, “Jamie has PTSD because he was in an abusive relationship, but our family was also abusive. Jamie has been in therapy basically all his life and received treatment for depression and PTSD. We have only told one therapist that we are multiple. The therapist said that Jamie has a functional life and there are even signs that we are high functioning, so we were never given a label. She (the therapist) did not want to meet me.” Sarah believes that being multiple is not a diagnosis and that it does not need any treatment. She feels that their system is stable and they live a functional life. Perhaps the persons in the system need treatment, but not the entire system.

#### Vignette 4—Zvire

“We are 28; our body is female,” she explained. Zvire lives in a small town of an urban area. She is unemployed, currently living on disability benefits.

“It is difficult for the resident persons to be a ‘we’,” she stated. There are 11 people in the system, including three or four core members, four children (all boys), and two other people “who aren't really part of the system.”

Her system started when she was 17 years old: “We were not created by trauma. It started from being a singlet (one body, one person), but then, we lost our sense of self. The boundaries of self became less and less distinct, and we slipped into each other. (…) I was the original. Then my sister turned up. She and I used to be very close.” The system used to be a “multiplex” (a large “complex” system with many members) but has changed to a medium size now.

She stated that switches happen unexpectedly: “‘I want to use this body now,’ they would say, and shove clean out of the way.” She explained that there is a chair in the system who is “normally there,” and her presence is reassuring. This person can step in when someone in the system becomes potentially aggressive. Many new events have happened to the system recently, and as a result, its structure has changed. She reported that communication among the system members has become more difficult recently: “Our sense of self is very, very bad at the moment.” A few months ago, she came home and saw folded clothes in the house, but neither she nor anyone else in the system remembered who folded the clothes. “Maybe other little secrets happen as well,” she stated. She assumes that switching may be beyond her control, as if “your body isn't doing what you're doing.”

She stated that their body has autism and attention deficit and hyperactivity disorder (ADHD) and added that, since the age of 15, they have not been able to get quality mental health treatment for being multiple. She has seen many psychiatrists but feels that they usually reject her or retire in the end. She received treatments for ADHD (methylphenidate) and depression, but she did not find that they worked. “My brain does not react to meds,” she stated. She thinks that one of her therapists had the suspicion that she had DID but rejected carrying out an assessment for differential diagnosis because he thought this was a good coping mechanism for her; therefore, “he didn't want to take it away.” Nowadays, she experiences a great amount of stress as a result of living on benefits and hints that these difficulties in her life are connected to the difficulties within the system.

#### Vignette 5—marigold system

“I'm the host. I'm agender, because I don't like to be identified by gender. We've decided, it's better that I talk because I've been here the whole time,” a 22-year-old female voice introduced herself. She lives in a small town with family and currently studies psychology in college (this is a coincidence with Vignette 1—Dylan).

There are 28 members in her system, whom she records in an Excel spreadsheet. There are a few dominant ones, approximately six or seven, and they tend to front much more often than the others. Everyone has a name. Within the system there are three families. One of the residents has another subsystem. There are three children, and the older residents take care of them. She gradually has been gaining control over her system: “If *I* come out, they have to back down. They all know this is *my* body. I can tell people they can't come in. I can control this.” By now the system is organized. “It is like a student association, like fraternities or a family,” she stated. “We even have a constitution. It is 15 pages long, and everybody in the system has to sign it.” She can always remember what happened, but other members cannot remember what the other one said or did.

She explained that switching “just happens,” especially under high stress, which she finds helpful. Triggers are different for different people. But, she stated, “if I ask, they would just come in. Switching happens on invitation. I can completely switch out, and I'm always there. I can pull them back.”

The system started about seven years ago, when the body was 15 years old. “We are not traumagenic; it just happened,” she stated. “In the very beginning, there were only two people who argued. One day I woke up and felt as if someone was possessing me. First I thought that I was losing my mind. It was crazy.” Then, a couple of months later, another person switched in, but it did not communicate with her. A little later, someone else came in and established order. Then, more switched in. There are fairly original members, and there are other ones who gradually showed up.

Her parents know about the system, but they have never met other members. “My mum asked me if I wanted to kill her,” she stated. Her teachers do not know about her system because she would never allow switching in the classroom. She talked to professionals, and they do not think it is a problem; they see it as a sort of help. Given that she has not had trauma or amnesia, they do not believe it is DID. She added, “It isn't interfering with my life now. On the contrary, it is helpful.”

#### Vignette 6

##### Phottae system—ethan

A 29-year-old female voice introduced herself: “I'm Ethan. Our body is female. I'm personally a male.” She lives in a rural town with her parents and a partner, who is also a system. She studies psychology and has plans to get a PhD and perhaps pursue the profession of counseling in the future.

She explained that there are 20 individuals in the system who are very separate mentally and mixed in terms of gender. There is no core person or host, but they all consider themselves equal. The one who is in the front controls the body. The others remain in the background, as if being asleep. There is constant and very good communication among them. They all have different colors, physical appearance, different opinions, beliefs, and things they are do not do well. They also have colored synaesthesia, their thoughts are colored and numbers appear as colors as well. Ethan believes he is blue. They try to match activities to talents. For example, those members who are good at cooking will cook. They think of this as a kind of superpower.

The front member did not always have full control of the body in the past. Sometimes the front, for example, felt as if the body's limbs were not hers (his), and the body bumped into things, was quite clumsy and had disastrous balance issues. Since multiplicity, she explained, they are able to reconnect better and are “pretty well-integrated.” Now, only one of them is fronting at a time, and thus, movement coordination is much more precise.

Switching occurs when the one who is fronting takes a step back and falls into a kind of sleeping state. They can hear each other's thoughts, but she emphasized that they know that those are not hallucinatory. Sometimes they also have meetings to discuss matters. She insisted that the body is shared “property,” and they all take good care of it.

She thinks that their biological mother might also be a multiple because she recalls her mother changing her mood suddenly and frequently.

They have been writing a joint journal for 15 years that they share. In retrospect, she recalled that journal entries were color-coded already during primary school, which she considers proof for the early existence of their system. Seven years ago, they started hearing each other's voices, so they went to see a doctor. They (she) were told that she had acoustic hallucinations. The diagnosis of narcolepsy emerged because there were alterations in wake-state brain waves. The first memories about switching between members are from the time when she was three or four years old. She stated that the system has a long history, but it was only given a name three and a half years ago when the multiplicity “officially” emerged.

She stated they had a mixed experience with doctors and psychiatrists. She stated that they were given medication and that her quality of health decreased. She was anxious, and they lost communication with one another. She tried to be “normal” but felt disabled instead, mainly because of the medication she had been given. She was on an experimental drug for three and a half years when she decided to stop taking it. She has even lost some memories while being on drugs. She reported that it took them more than two years to recover and become aware of each other again.

She explained that she has been seeing a therapist for 3 months now to address her anxiety, which she often feels. Her therapist knows about the multiplicity and accepts it. She believes that multiplicity, as long as it is adaptive, does not require treatment. Only memory issues, anxiety or anger among the members are features that may need treatment. The “Phottae system” is married to another multiple system. They met in college.

At the end of the interview, we asked if another interview could be conducted with another member of the system. A few days later, Notarovych contacted us and volunteered for the interview.

##### Phottae system—Notarovych

Notarovych opened the conversation: “I believe you talked to my system mate Ethan the other day.” She had a female voice that sounded slightly different from Ethan's voice. “Ethan is an extrovert, unlike most of us in the system,” she added.

She stated: “I agree with Ethan that we are proficient in switching in order to have the appropriate person at the appropriate time. In our system, there are about six to eight members. All of us had been placed here in this body before we were born, and all of us were placed in the body for a purpose. I think we were born multiple. I have been here for as long as we can remember. I have pictures that I drew when I was five or six years old. We have intense dreams, and dreams can be a place to meet others for the first time. Our dreams are vivid and lucid. The dreams are not synesthetic, but colorful and spiritual. I am the only one in the system who can control and change the dreams. When we fall asleep, someone starts fronting in the dream, then falls out of the dream, and someone else falls in the dream. I can step in during a dream and take the lead.”

She usually “puts people in their place,” and then, she leaves in order not to get hurt. She also does the same with some system members. She is a pagan and fights back if others (the atheists) pick on her because of her religion. “In the past, I was reluctant to work together with other system members, but I realized it was easier for all of us to do so,” she stated. “They allowed me to practice my faith, and in return, I can be brave in situations that they would be afraid of. I think this is a good deal.”

“We have food allergies and celiac disease; we eat gluten-free,” she stated. She does not drink alcohol, as the body is quite sensitive to it and falls asleep as a result. While she was on the medication, she felt that she was pushed down. She explained that the medication was too much, so she sabotaged it. She stated that she told everyone things to mislead them on purpose and to prevent the worst diagnosis. She feels much better being off the medications now and feels that her spirituality has returned.

### Summary of interviews

The interviewed persons were typically in their early 20 s, and all were female at birth. The number of persons in each system ranged between 2 and 28. Some alters (resident persons) are completely equal to one another, while others are hierarchical in their structure and may even have subgroups within the larger system. Often, one alter controls the behavior at a time, which is called fronting. Alters have their own names, histories, and personalities, and they often have their own ways of communicating and behaving. Switching—when an alter becomes the fronting person—may happen under stress and involuntarily, but it also may happen consciously, depending on the level of control within the particular system. Please see Table 1 for a summary of system characteristics.

**Table 1:** Summary of the Interviewed Multiples.

	**1**	**2**	**3**	**4**	**5**	**6**	
**Name or pseudo-name**	Dylan (Kerry and Wotany)	Space system (Matthew)	Sarah and Jamie	Zvire	Marigold system	Phottae system (Ethan)	Phottae system (Notarovych)
**Age**	19	23–24 (estimated)	23	28	22	29	
**Gender (of the body)**	Female	Female	Female	Female	Female body, but considers herself to be agender	Female body, but Ethan is male	Female
**Number of personalities in the “system”**	9	19–20	2 (brother and sister)	11	28	21	
**Age of onset**	12–13 years	6	Cannot remember	NR	15	Memories of switching from 3 to 4 years	“since before birth”
**Diagnosis**	DID	Major depr., GAD, PTSD, DDNOS, but believes she has DID	Depression, PTSD, eating disorder	ADHD, major depression, autism	Professionals do not think it is a problem	Narcolepsy (brain waves changed in sleep), has seen a therapist for anxiety	
**Control over switching**	Partial. Switching happens as a result of stress	Both conscious and unconscious switching	Decides when to switch; both follow a schedule	Beyond control, someone “comes forward”	complete	NR	NR
**Awareness of other alters in the system**	Mostly	Complete	Complete (although memory feels “different”)	“little secrets happen”	Host can always remember, but they cannot remember each other	These days, she remembers, but memories have “different feelings”	NR
**Comment**	Usually does not remember what happens when another person is fronting	Lives like tenants in a large house, with one “landlord”	Was on hormones for a while (female to male)	Clinicians rejected the diagnosis of DID because they thought it was a good coping mechanism	People are listed in an Excel file, three families, who have a constitution		Pagan

*Names were changed to protect their identities. NR, not reported; ADHD, attention deficit and hyperactivity disorder; DID, dissociative identity disorder; GAD, generalized anxiety disorder; PTSD, post-traumatic stress disorder; DDNOS, dissociative disorder not otherwise specified*.

The existence of the system often dated back to childhood and, in some instances, even to the earliest memories. Certain cases appeared to exist as a result of trauma, but in most cases, there did not seem to be any preceding traumatic event. The condition appears to be resistant to antipsychotic medications, except in one case. All six cases found the existence of the term and the concept of multiplicity helpful. They also found that online forums were a place where they could encounter other multiples and interact in a helpful way. Multiplicity and the possibility for online socializing appear to be a way of coping, which results in a relatively better level of functionality.

There are several notions that support the idea of an extreme form of identity complexity, whereas others support the notion of a dissociative disorder. Nevertheless, group identity and the possibility for interaction provided by multiplicity forums seem to help members understand and accept their experiences, thus improving their ability to cope.

## Discussion

**Figure 1:**
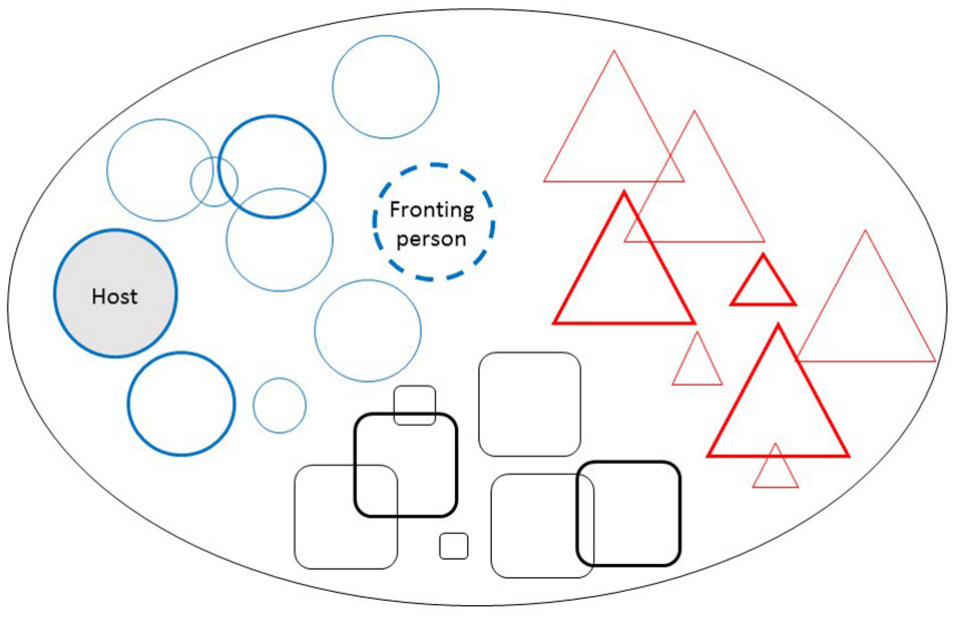
A Typical Personality Organization of a Multiple. In this example, there are 25 resident persons in the system, and each person is a separate self. There are three families (subsystems). There is one host, who is the “main” person, usually the first to be present in the system or the body (shown in the plain circle); one fronting person, who organizes the behavior (shown as a dashed circle); dominant members (shown with greater weighted outlines); and children (small shapes). Each member has a different name, preferences, behaviors, sexual orientation and gender or can be agender.

The current study presented the cases of six people who consider themselves to be a multiple: a system of alternative personalities sharing the same physical body. This is the first study to report on experiences through personal interviews of multiplicity. People who identify with this group believe that, instead of having one self with altering mood states and behaviors, there are several distinct selves, each having their own unique behavioral pattern (see Figure 1). Personality is not the “mean” of the different behaviors that are triggered by different situations, but instead, there is an overarching personality, called the “system,” which may consist of a group of individual selves or member personalities. Multiples refer to themselves as “we,” instead of “I.” Multiples can be placed along a continuum between identity disturbance and DID, although most systems function relatively well in everyday life, thus casting doubt over the presence of a pathology.

Our findings support the notion that multiplicity is a social construct where identities are established and maintained through social interaction and follow rules supporting the concept of multiple personality disorder by Spanos (1994; see also Gleaves, 1996). From this perspective, multiplicity or plural self is the “modern” manifestation of a minority of people who tend to develop several selves (i.e., in the form of spirit possession or by being highly susceptible to social cues of “having” more than one personality); thus, multiplicity is a social creation (Spanos, 1983). Nevertheless, the clear presence of dissociation in our study (i.e., change of handwriting) renders multiplicity a more heterogenous concept than previously considered.

### Multiplicity and dissociative disorders

According to DSM-5 (American Psychiatric Association, 2013), depersonalisation occurs when one experiences unreality or detachment from one's mind, self or body, whereas experiences of unreality or detachment from one's surroundings is called derealisation. In multiplicity, depersonalisation occurs in the form of detaching the self from other possible selves and making it dominant; thus, multiplicity can be considered a special form of depersonalisation. However, “multiple systems” generally have a clear sense of reality. Therefore, derealisation may only be episodic, if it occurs at all. This study did not find traces of dissociative amnesia; that is, memories usually were retained normally in parts of the self and could be recalled. Autobiographical information was intact, although not every alter shared every memory event. Dylan, for example, had signs of involuntary changes in handwriting and “losing time” (note that she had received the diagnosis of DID). Others, like Marigold, kept full control of their alters and decided which member received the opportunity to front, without having amnesia.

The idea of DID was only partially supported. Disruption of identity was clearly evident in a way that continuity in identities (alters) in the sense of self, agency, and behavior was intact, which was acknowledged by others (often by professionals), as well. Furthermore, memories were available (especially where switching was under voluntary control), and everyday functioning seemed to be relatively intact, which contradicts the idea of DID.

### Extreme form of possible selves

In many narratives, the presence of alters (alternative selves) appears to be a coping mechanism for past traumas and daily stress. Coping happens through the splitting and personalizing of different personality parts, such as “the aggressor” or “the motherly protector.” This is similar to cognitive theories of the self, such as the possible selves that are “the cognitive components of hopes, fears, goals, and threats, and they give the specific self-relevant form, meaning, organization, and direction to these dynamics” (Markus and Nurius, 1986, p. 954.). In addition, multiplicity can be regarded as an extreme form of self-complexity, in which the increased number of self-aspects are protective against negative affections and may serve as a buffer against the adverse impact of stress on depression or other mental illness (Linville, 1987). This theory is supported by the fact that those who can switch (i.e., among alternative personalities) generally report better functioning compared to those who are unable to control the switches. Both the theory of possible selves and of self-complexity predict that the greater number of selves (or self-aspects), the better they function. This is supported by the notion that multiples generally report a better sense of well-being than before they are aware of the community and identify themselves as multiple.

### The role of group membership

Systems generally like to give a narrative and describe their resident persons, their preferences, interests and dislikes in great detail. Defining themselves in the search of stable identities appears to be an ongoing process for many systems. It is remarkable how the common identity of “being multiple” aids in the process of coping with the alterations of the personality. Clearly, because of the online community and frequent interactions, people who consider themselves multiple begin to use common terminology and construct their own reality in ways similar to one another. Thus, they influence each other and share experiences, which leads to a shared identity (Edwards and Middleton, 1986). This is made possible through shared discourses on online multiplicity forums, similar to personal narratives in Alcoholics Anonymous. Thus, individual and collective experiences are merged into the same therapeutic process (Steffen, 1997). Furthermore, it has been demonstrated previously that identification with a minority group suppresses the negative effect of prejudice on self-esteem (Branscombe et al., 1999; Schmitt et al., 2003); thus, the label of multiplicity might be helpful in coping with unusual sensations and negative external feedback. This is supported by the subjective experience of the interviewed individuals, who all report that the discovery of the concept of multiplicity and the possibility of communicating with others was helpful and therapeutic.

However, unlike self-help groups, these individuals did not gather in order to recover. The notion behind multiplicity forums is to share and, ultimately, maintain the behavior (as well as support each other). For this reason, the existence of online forums for multiplicity is not without any risk. On the one hand, the online community may prevent members seeking professional help, and on the other hand, individuals with disturbed but not dissociated identity problems also may internalize the group's beliefs and rules, further increasing the severity of their fragmentedness.

### Biological traces

Vignette 1 (Dylan) received a diagnosis of DID, whereas Vignette 2 (Space system) was diagnosed with PTSD. They both have found selective serotonin reuptake inhibitor (SSRI) medication helpful. However, neither Vignette 4 (Zvire), who was diagnosed with autism and ADHD, nor Vignette 6 (Phottae), who was diagnosed with anxiety, have benefitted from antidepressants or any other medication they received. The latter system also had colored synaesthesia, and she reported that her mother probably had DID. Therefore, it appears that there may be several underlying biological abnormalities, possibly genetic proneness, although only the minority of multiples benefit from the prescribed medication. In line with this, previous studies have shown that the most frequently prescribed medication in DID and dissociative symptoms not otherwise specified are antidepressants (76%) and antianxiety medications (74%; Brand et al., 2009), although medications may only reduce the symptoms (e.g., nightmares or aggression) and cannot “cure” dissociation in the long term (Gentile et al., 2013).

### Limitations of the study

The current study is not without limitations. Given the nature of the data collection, information was unable to be gathered from people other than multiples, such as from their relatives or friends. Furthermore, due to the limited time for interviews, limited information was obtained about multiples' lives outside the Internet, therefore, preventing a full assessment of functionality. In addition, interviews were voluntary and relied on self-reporting of experiences, which might have led to a biased sample and assessment. Given the lack of questionnaires, future studies should focus on developing a reliable questionnaire to assess multiplicity and to obtain quantitative, comparable data.

### Conclusion

With the increasing popularity and spread of the Internet, various forms of self-organized support groups have emerged. Multiplicity is a relatively new concept that encompasses people who consider themselves multiple by nature; that is, they have a group of individual selves who share the same body. It can be concluded that multiplicity is a label and a self-organized support group for people with severe identity disturbances, in some cases with symptoms of dissociative disorders. Further research is needed to assess clinically the underlying motivations, functionality and long-term changes in individuals who consider themselves multiple.

## Ethics statements

Institutional Review Board of Eotvos Lorand University Consent was sought prior to conducting the interviews. Participants were informed about the study and agreed to take part and the information to be reported in a scientific paper anonymously.

## Author contributions

GR, ZD, and AM designed the study. GR, AM, and LL contributed to data collection. All author wrote, revised and approved the final manuscript.

### Conflict of interest statement

The authors declare that the research was conducted in the absence of any commercial or financial relationships that could be construed as a potential conflict of interest.
