# Supplementary material for: Multiplicity: An Explorative Interview Study on Personal Experiences of People with Multiple Selves
Source: Front Psychol. 2017 Jun 13;8:938. doi: 10.3389/fpsyg.2017.00938 (PMC5468408; doi:10.3389/fpsyg.2017.00938)
Supplement: Supplementary file 1 [file DataSheet1.DOCX]

**Appendix 1**

**Semi-structured interview questions used to assess multiplicity.**

1. Demographic information (gender, age, education)
2. Previous psychiatric treatment, clinical diagnoses
3. The nature of multiplicity:

- how many “resident persons” in the body?

- do “resident persons” know about each other?

- what leads to switching between resident persons?

- illness insight

1. Dissociative Identity Disorder (similarities and differences) – any previous DID diagnosis?
